# Highlight: “Jumping Genes” Caught in an Aging Merry-Go-Round

**DOI:** 10.1093/gbe/evaf094

**Published:** 2025-06-02

**Authors:** Pedro Andrade

No one escapes aging. This is such a fundamental biological process that it may surprise how little we still know about what causes aging and how it evolved. Aging can be broadly defined as the decline in an individual's reproductive and survival capacity through time; from an evolutionary perspective, it is a consequence of relatively stronger selection on genetic variants that benefit early-life fitness (Rose 1991, Li et al. 2023). Multiple theories have been proposed to explain both the selective constraints that allow aging to occur and the mechanisms underlying it. An important aspect of aging is that it is a characteristic of a given species; even accounting for individual variation, the aging trajectory within a conspecific group of individuals is broadly similar. This means that, to some extent, aging is encoded in the genomes of organisms.

Transposable elements (TEs), a ubiquitous feature of the genomes of both eukaryotes and prokaryotes, have been implicated in aging. These virus-like mobile genetic elements can comprise the majority of the genetic information of an organism and are increasingly recognized as drivers of functional evolution (Bourque et al. 2018). According to Cristina Vieira—professor at the Université de Lyon, in France—TEs are essential to understand aging due to their ability to reshape the genome: “their activity can compromise genome stability, modulate gene expression, disrupt regulatory networks, and produce epigenetic modifications, all of which are hallmarks of aging” says Vieira. But does the genome-jumping activity of TEs promote aging? Or is it the other way around, with TEs functioning as a byproduct of the inevitable senescence of individuals? According to Vieira and coauthors (Merenciano et al. 2025) in a new Review in *Genome Biology and Evolution*, it can be both!

In their review, the authors provide an extensive description of how TEs are associated with aging across lineages and among tissue types. On one hand, aging promotes increased expression of TEs, making these a reliable predictor of organism age. The root causes of this are, however, not clear. On the other hand, TEs by themselves also promote aging; as they move through the genome, TEs can insert themselves in genomic locations of functional significance, thereby contributing to reduced longevity. These movements end up causing genomic instability (e.g. through double-strand breaks), ultimately contributing to a series of diseases, including Alzheimer's disease, amyotrophic lateral sclerosis, and several types of cancers. Hence, the genomic presence of TEs can be both a cause and a consequence of aging (Fig. 1).

**Fig. 1.**
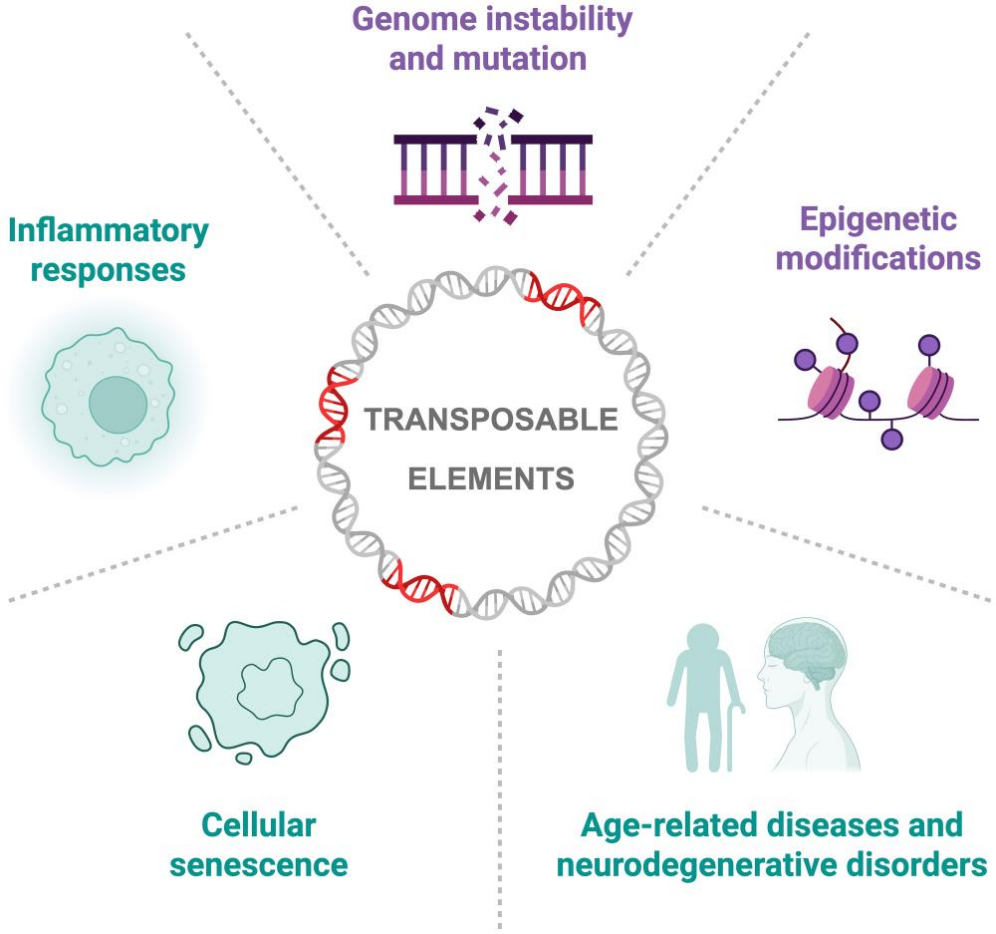
TEs are at the center of the aging process as both cause and consequence of time-related deterioration of vital physiological and cellular functions (illustration by Miriam Merenciano).

Some of these aging–TE relationships are particularly well exemplified by the authors' comparisons of organisms with distinct aging patterns. Sex-related differences in longevity, for example, are associated with increased TE activation in the sex-restricted heterochromatic chromosomes, such as the mammalian and *Drosophila* male Y chromosomes. Also, long-lived organisms tend to have signatures of reduced TE activity, such as the iconic *Hydra*, a potentially immortal self-regenerating cnidarian that shows lifelong activity of the TE-repressing PIWI-interacting RNAs (piRNA) pathway.

But are TEs the harbinger of our inevitable physical decline? Not necessarily. Merenciano et al. (2025) also cite research showing how TEs can contribute to longevity, either by initiating coordinated cell death processes that can suppress tumors or by facilitating the elongation of telomeres at the distal ends of chromosomes. A striking example of how a higher genomic TE content does not equal reduced longevity is the Greenland shark (*Somniosus microcephalus*), the longest-lived vertebrate, whose genome comprises an impressive 70% of TEs.

The TE–aging relationship is thus far from straightforward. Improving its understanding would need, as Vieira explains, a more detailed functional exploration of these genomic elements. “Since transposable element activation can vary between different cell types and tissues, single-cell transcriptomic and epigenomic profiling are good techniques to identify where and when specific TEs become active, and how these events correlate with changes in chromatin state or gene expression” says Vieira, who adds: “functional manipulation of transposable elements through CRISPR-based approaches, for example, can offer a direct way to assess their causal role in aging phenotypes as well.”

Another recently published article in this journal underscores how aging is also associated with other cellular and physiological markers. Hubert et al. (2025) focused their attention on how the metabolome correlates with differences in aging. These authors followed an experimental evolution approach to produce replicate *Drosophila melanogaster* populations independently selected for an accelerated aging phenotype. Then, they profiled the metabolomes of these fast-aging fruit flies to understand how selection shapes their physiology. These authors found that the metabolome was a repeatable and predictable marker of aging and that specific metabolites are associated with the aging process (Hubert et al. 2025). In particular, they highlight differences in energy substrate use with age, since accelerated aging fruit flies showed decreases in metabolites associated with glucose processing. Beyond metabolomics, their findings demonstrate the potent force of selection in shaping phenotypes through convergent evolution.

These advances show the considerable progress that has been made in understanding the evolutionary history and mechanisms of aging; yet there is much to be learned. Untangling this complex phenomenon will require further comparative studies from the genomes of diverse lineages. As Vieira adds, in regards to TEs, “future research should aim to clarify the role of TE activation in aging across different species and sexes, especially given the well-documented sex differences in lifespan. Additionally, investigating how TEs interact with epigenetic regulators during aging holds great potential for the development of novel therapeutic interventions, where targeted modulation of TE activity could help alleviate age-related dysfunctions.” Understanding these complex phenomena will require further comparative studies based on genomes from diverse lineages.

**Want to learn more?** Check out these other articles on the evolution of TEs recently published in *Genome Biology and Evolution*:

- Ancona L, Nitta Fernandes FA, Biello R, Chiocchio A, Castrignanò T, Barucca M, Canestrelli D, Trucchi E. Evolutionary dynamics of transposable element activity and regulation in the Apennine yellow-bellied toad (*Bombina pachypus*). *Genome Biol Evol.* 2025:17(4):evaf062. https://doi.org/10.1093/gbe/evaf062.
- Mallik R, Wcisel DJ, Near TJ, Yoder JA, Dornburg A. Investigating the impact of whole-genome duplication on transposable element evolution in teleost fishes. *Genome Biol Evol*. 2025:17(1):evae272. https://doi.org/10.1093/gbe/evae272.
- Oliveira JI, Lane C, Mugambi K, Yildirir G, Nicol AM, Kokkoris V, Banchini C, Dadej K, Dettman J, Stefani F, Corradi N. Analyses of transposable elements in arbuscular mycorrhizal fungi support evolutionary parallels with filamentous plant pathogens. *Genome Biol Evol*. 2025:17(3):evaf038. https://doi.org/10.1093/gbe/evaf038.

## References

[evaf094-B1] Bourque G, Burns KH, Gehring M, Gorbunova V, Seluanov A, Hammell M, Imbeault M, Izsvák Z, Levin HL, Macfarlan TS, et al Ten things you should know about transposable elements. Genome Biol. 2018:19(1):199. 10.1186/s13059-018-1577-z. 30454069 PMC6240941

[evaf094-B2] Hubert DL, Arnold KR, Greenspan ZS, Pupo A, Robinson RD, Chavarin VV, Barter TB, Djukovic D, Raftery D, Vue Z, et al Selection for early reproduction leads to accelerated aging and extensive metabolic remodeling in *Drosophila melanogaster*. Genome Biol Evol. 2025:17(5):evaf082. 10.1093/gbe/evaf082. 40326415 PMC12093319

[evaf094-B3] Li S, Vazquez JM, Sudmant PH. The evolution of aging and lifespan. Trends Genet. 2023:39(11):830–843. 10.1016/j.tig.2023.08.005. 37714733 PMC11147682

[evaf094-B4] Merenciano M, Larue A, Garambois C, Nunes WVB, Vieira C. Exploring the relationship of transposable elements and ageing: causes and consequences. Genome Biol Evol. 2025:17(6):evaf088. 10.1093/gbe/evaf088. PMC12127037 40373205

[evaf094-B5] Rose MR . Evolutionary biology of aging. New York (NY): Oxford University Press; 1991. p. 221.

